# Metformin promotes the survival of transplanted cardiosphere-derived cells thereby enhancing their therapeutic effect against myocardial infarction

**DOI:** 10.1186/s13287-017-0476-7

**Published:** 2017-01-28

**Authors:** Rongchuan Yue, Wenbin Fu, Xiang Liao, Cong Lan, Qiao Liao, Liangpeng Li, Dezhong Yang, Xuewei Xia, Xiongwen Chen, Chunyu Zeng, Wei Eric Wang

**Affiliations:** 1 Department of Cardiology, Daping Hospital, Chongqing institute of Cardiology, Third Military Medical University, 10 Changjiangzhilu Road, , Yuzhong District Chongqing, 400042 China; 2 Department of Cardiology, Chuanbei Medical College, Sichuan, 637007 China

**Keywords:** Metformin, Cardiosphere-derived cells, Myocardial infarction, AMP-activated protein kinase

## Abstract

**Background:**

Transplantation of cardiosphere-derived cells (CDCs) has been shown to exert a therapeutic effect in patients with myocardial infarction (MI). However, poor survival of transplanted CDCs limits their beneficial effect. Metformin (MET) activates AMP-activated protein kinase (AMPK) which is associated with cell survival. The aim of this study is to determine whether MET improves CDC survival in the transplantation microenvironment and enhances the therapeutic effect of CDC transplantation against MI.

**Methods:**

CDCs were isolated and expanded from transgenic β-actin-GFP mice. CDCs were pretreated with MET and intramyocardially injected into wild-type C57 mouse heart with MI injury. The survival of CDCs was quantified, and the infarct size and cardiac function of treated hearts were evaluated.

**Results:**

CDC transplantation modestly reduced infarct size and improved cardiac function in the post-MI heart, which was further improved by MET treatment. MET pretreatment significantly increased the survival of CDCs transplanted into the myocardium. MET also reduced CDC apoptosis induced by oxidative stress in vitro. The anti-apoptotic effect of MET was blocked by the AMPK inhibitor compound C. MET increased AMPK phosphorylation and upregulated endothelial nitric oxide synthase (eNOS) in CDCs under oxidative stress, which might be associated with the anti-apoptotic effect of MET.

**Conclusions:**

MET improves the survival of transplanted CDCs in the myocardium, thereby enhancing their therapeutic effect against MI injury. The pro-survival function of MET on CDCs might be associated with an AMPK-eNOS-dependent mechanism.

**Electronic supplementary material:**

The online version of this article (doi:10.1186/s13287-017-0476-7) contains supplementary material, which is available to authorized users.

## Background

Myocardial infarction (MI) is a major severe heart disease that threatens human health worldwide. The substantial loss of functional cardiomyocytes contributes to the pathogenesis of the disease, leading to cardiac failure. Stem cell therapy offers possibilities for restoring lost myocardium and cardiac function. Multiple cell types, including bone marrow-derived cells and adipose tissue-derived cells, have shown modest therapeutic effect on post-MI patients [1]. Another cell type which has been applied to clinical trials is cardiosphere-derived cells (CDCs), which showed protective and regenerative capacity in both post-MI animal models and patients [1, 2]. Autologous CDCs decreased scar size, increased viable myocardium, and improved cardiac function in patients [1]. Transplantation of embryonic stem cell- or induced pluripotent stem cell-derived cells has shown promising effect in post-MI animals [3, 4], although risks of tumorigenesis and arrhythmia currently limit their application in patients.

Irrespective of the cell types used for transplantation, effective cardioprotection and cardiac muscle regeneration are hindered by the poor survival of transplanted cells in the infarcted myocardium, which is high in inflammatory factors and free radicals [5]. Genetic manipulation of pro-survival genes such as prolyl hydroxylase domain protein 2, Akt, Bcl-2, SDF-1, and chemokine receptor 1 have improved the viability of transplanted stem cells in animal studies [6], but these gene targets are difficult to interfere with in the clinical setting thus far. An alternative approach is to identify a known medicine, which has been used in clinics, to improve the stem cell survival thereby enhancing the therapeutic efficacy.

Metformin (MET), a first-line antidiabetic medication for the treatment of type 2 diabetes, has been reported to inhibit the mitochondrial respiratory chain and activate AMP-activated protein kinase (AMPK). AMPK can stimulate fatty acid oxidation, promote glucose transport, accelerate glycolysis, and inhibit both triglyceride and protein synthesis. AMPK stimulation promotes cardiomyocyte cell survival through a mitochondrial apoptotic mechanism [7]. MET attenuates oxidative stress-induced cardiomyocyte apoptosis and prevents the progression of heart failure in dogs, through a mechanism of activating AMPK [8]. MET also exerts a cardioprotective effect against doxorubicin-induced cardiotoxicity [9, 10]. Furthermore, MET is found to attenuate pulmonary inflammation in animals [11]. Given the anti-apoptotic effect and the potential for reducing inflammation, we here test if MET increases the survival of stem cells in the harsh transplantation environment of the ischemic heart. In the present study, MET treatment was combined with CDC transplantation in the post-MI mouse hearts. The therapeutic effect against MI injury was evaluated, and the underlying mechanisms were further explored.

## Methods

### Ethics statement

Male wild-type C57 mice were purchased from the Laboratory Animal Center of Daping Hospital, and transgenic β-actin-GFP mice (C57BL/6-Tg (CAG-EGFP) C14-Y01-FM131Osb) were purchased from the Jackson Laboratory, USA. All experimental procedures using the animals were carried out in accordance with the regulations of the Animal Care and Use Committee of the Third Military Medical University, China. All animal protocols were approved by the Medical Ethics Committee of the Third Military Medical University (Permit Number: 2012[13]). All efforts were made to minimize animal suffering.

### Culture and characterization of mouse CDCs

Mouse CDCs with transgenic green fluorescent protein (GFP) labeling were obtained and expanded with a protocol similar to that previously reported [12]. GFP labeling was used for tracing the cell fate of CDCs post-transplantation. Briefly, heart tissues of 4-month-old transgenic β-actin-GFP mice were cut into fragments <1 mm^3^, washed, and partially digested with trypsin (0.05%; GIBCO). These tissue fragments were cultured as cardiac explants on fibronectin-coated (20 mg/mL; Sigma) dishes in 2 mL cardiac explant media (CEM; Iscove’s modified Dulbecco’s medium (IMDM) supplemented with 20% fetal bovine serum (FBS) and 0.1 mmol/L 2-mercaptoethanol). After a variable period of growth, the loosely adherent cells surrounding the explant (cardiac outgrowth) were harvested using mild enzymatic digestion (0.05% trypsin/EDTA) and dispensed to poly-d-lysine-coated dishes. Several days later, cardiospheres were harvested, plated on fibronectin-coated flasks, and cultured in cardiosphere growth medium (CGM; Dulbecco’s modified Eagle’s medium (DMEM)/F12 supplemented with 35% IMDM, 7% FBS, 29.2 mg/mL l-glutamine, 0.1 mmol/L 2-mercaptoethanol, 2% B27 supplement, 25 ng/mL cardiotrophin, 20 ng/mL basic fibroblast growth factor (bFGF), 10 ng/mL epidermal growth factor (EGF), and 5 U thrombin) to generate CDCs. The characterization of CDCs was investigated by flow cytometry. CDCs were incubated with antibodies against CD29, CD105, CD90, CD31, CD45, and CD34 for 30 min. Isotype-identical antibodies served as a negative control. Quantitative analysis was performed using a BD Accuri™ C6 Cytometer and FlowJo software.

### Mouse MI model and intramyocardial injection of CDCs

Animals were randomly allocated into groups with different treatments. The mouse MI model was performed in 4-month-old wild-type C57 mice according to the methods we described previously [6]. Under anesthesia with isoflurane inhalation, permanent ligation of the left anterior descending (LAD) coronary artery was made, and CDCs were injected intramyocardially. The LAD artery was permanently ligated with 7-0 silk suture under direct vision. Sham-operated mice were subjected to the same surgical procedures except with a tied suture. Using a similar protocol to that previously reported [13], MET was intraperitoneally injected once a day at a dosage of 125 mg/kg for 7 days (the first dose 6 h before MI injury). For cell transplantation, C57 mice were subjected to an intramyocardial injection immediately after MI injury at four points in the infarct border zone with 40 μL phosphate-buffered saline (PBS) containing 1 × 10^5^ CDCs. MET was used to pretreat CDCs (dissolved in PBS at 100 μmol/L containing the CDCs before transplantation) and intraperitoneally injected into the recipient animal.

### Echocardiography analysis

Echocardiography was performed (GE vivid 7 dimension) to determine cardiac structure and function in anesthetized mice. Mice underwent echocardiography at 4 weeks after surgery. After the induction of anesthesia by inhalation of isoflurane, the hearts were imaged two dimensionally (2D) in long-axis views at the level of the greatest left ventricular (LV) diameter. Left ventricular ejection fraction (LVEF) was automatically calculated by the echocardiography software using Simpson’s method. EF% was calculated as ((LVVd – LVVs)/LVVd) × 100, where LVVd is left ventricular end-diastolic volume (μL) and LVVs is left ventricular end-systolic volume (μL). Left ventricular fractional shortening (FS) was calculated as ((LVDd – LVDs)/LVDd) × 100, where LVDd is left ventricular end-diastolic dimension (mm) and LVDs is left ventricular end-systolic dimension (mm). Echocardiographic data were conducted and analyzed by blinded investigators.

### Quantification of CDC survival

To quantify the survival rate of post-transplanted CDCs, heart cells were isolated from the mouse heart at different time points after CDC transplantation using a method of enzymatic digestion [6]. Briefly, heparinized animals were anesthetized and the hearts were rapidly mounted to a langendorff apparatus. The hearts were perfused retrogradely with Ca^2+^-free normal Tyrode’s solution containing 1 mg/mL collagenase II (Roche) and 0.1 mg/mL protease (Sigma) for about 10 min, depending on digesting conditions. The ventricles were cut off and minced in solution, followed by pipetting to disassociate the cells, and then filtered through a nylon mesh to remove big pieces of undigested tissues. The GFP^+^ cells represented CDCs or their derived cells, which were carefully counted. The survival rate = 100% × (number of survived GFP^+^ cells/1 × 10^5^).

### Inflammatory cell infiltration analysis

Similar to the method previously described [6], mouse hearts in the different groups were digested at 48 h after MI. Using a 30-μm filter, the small cells (<30 μm) were collected while most of the cardiomyocytes (>30 μm) were discarded. Inflammatory cell populations were analyzed by BD Accuri™ C6 Cytometer. Representative contour plots were made showing infiltrating monocyte cells in the heart, as defined by the expression of Ly6C^high^ and CD11b^+^. Data were analyzed by FlowJo software.

### Histology

Hearts were fixed with 4% paraformaldehyde, subsequently embedded in paraffin, and cut to 5-μm thick sections. Similar to the method previously described [14], quantitative morphometric analysis with Masson’s trichrome staining was performed. The area of infarct size was calculated by dividing the midline length of the infarcted LV wall by the midline length of total LV wall [15]. The percentage of the fibrotic area of the left ventricle was measured using Image J software. Individuals conducting the experiment were blinded to the experimental groups. To observe the survival of GFP^+^ CDCs in hearts, anti-GFP, anti-sarcomeric tropomyosin (Sigma), and DAPI for nuclei were used to stain the heart tissue. To trace CDC differentiation, immunostaining against GFP, sarcomeric tropomyosin (a cardiomyocyte marker), von Willebrand factor (vWF; an endothelial cell marker), and α-smooth muscle actin (α-SMA; a smooth muscle cell marker) were performed.

### The assessment of CDC survival in vitro

CDCs were cultured in serum-free media for 24 h and H_2_O_2_ (30 μmol/L for 24 h) was used to induce CDC apoptosis. CDCs were pretreated with MET at different dosages (1, 10, 100, 1000, 3000, or 5000 μmol/L) and for different time periods (15, 30, 60, 120, and 180 min) for inhibiting H_2_O_2_-induced CDC apoptosis. Administration of MET at 100 μmol/L for 60 min was selected for the experiment. AICAR (500 μmol/L for 60 min) was used to activate AMPK, and compound C (CC; 20 μmol/L for 4 h) was used for inhibiting AMPK [8]. Cell viability was detected using the CCK8 Cell Proliferation Assay Kit (Dojindo). CCK8 reagent (10 μL) was added to each well and incubated for 2 h at 37 °C. The number of viable cells was calculated by absorbance measurements at 450 nm. For evaluating CDC apoptosis, the terminal deoxynucleotidyl transferase-mediated dUTP nick-end labeling (TUNEL) assay was undertaken. Apoptosis was also quantified by flow cytometry (FACScan; Dickinson and Company) after cells were stained with annexin V and propidine iodide (PI) by using an AnnexinV-FITC Apoptosis Detection Kit (Sigma) [6].

### Western blot analysis

Total cellular extracts were obtained by cell lysis, as described previously [6]. Briefly, aliquots of cell lysates containing 50 μg protein were separated by sodium dodecyl sulfate-polyacrylamide gel electrophoresis (SDS-PAGE). The protein was then transferred from the gel to polyvinylidene fluoride membranes, which were incubated with primary antibodies for 1 h at room temperature and then at 4 °C overnight. After washing with Tris-buffered saline Tween-20, the membranes were incubated with horseradish peroxidase (HRP)-conjugated secondary antibody diluted 1:5000 (Cell Signaling Technology) and the signals were detected by chemiluminescence. Quantification of the bands was carried out using Quantity One software. To ensure equal loading of the protein, GAPDH was used as an endogenous control.

### Reagents

1-Dimethylbiguanide hydrochloride (metformin hydrochloride), 5-aminoimidazole-4-carboxamide ribonucleoside (AICAR; an AMPK activator), 6-(4-(2-piperidin-1-yl-ethoxy)-phenyl)-3-pyridin -4-yl-pyyrazolo(1,5-a) pyrimidine (compound C; an AMPK inhibitor) and H_2_O_2_ were purchased from Sigma-Aldrich (USA). Antibodies against phospho-AMPK-Thr-172 and AMPK were purchased from Cell Signaling Technology. The antibodies against endothelial nitric oxide synthase (eNOS) and phospho-eNOS-Ser-1177 were purchased from Santa Cruz Biotechnology.

### Statistical Analysis

Data are reported as the mean ± SEM. For analysis of differences between two groups, a student’s *t* test was performed. For multiple groups, one-way analysis of variance (ANOVA) with post-hoc comparisons by the Tukey’s test was used. Data were analyzed using SPSS 13.0 for Windows (SPSS, Chicago, IL, USA). A *P* value <0.05 was considered significant.

## Results

### Characterization of cell phenotypes

**Fig. 1:**
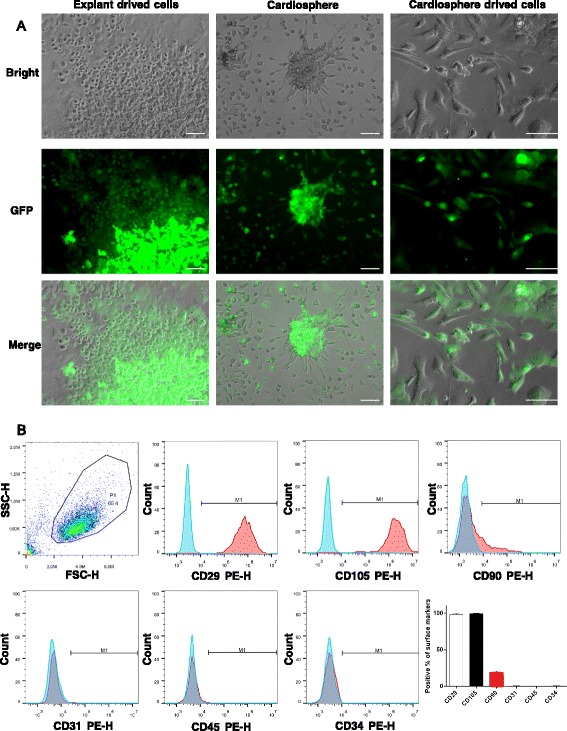
The culture and characterization of cardiosphere-derived cells. **a** Cells grew from the explant on top of a layer of stromal-like cells after 7 days. Cardiospheres formed after 4 days in suspension culture on poly-d-lysine-coated dishes. Cardiosphere-derived cells become confluent after 5 to 7 days. *Scale bar* = 100 μm. **b** Flow cytometric analysis showed that cardiosphere-derived cells were positive for CD29, CD105, and CD90, and negative for CD31, CD45, and CD34. *GFP* green fluorescent protein

CDCs were isolated and expanded from transgenic β-actin-GFP mice with a protocol similar to that reported previously [12]. GFP labeling was used to trace the CDCs after transplantation into the myocardium. As shown in Fig. 1a, the partially enzymatically digested ventricular explants spontaneously yielded outgrowth cells (explant-derived cells). These cells were harvested and formed three-dimensional cardiospheres. Subsequent replating of cardiospheres on adherent culture dishes yielded CDCs which were expanded and used for the experiments. Flow cytometry analysis revealed that CDCs expressed CD29 (98.1%), CD105 (99.2%), and CD90 (19.6%), but were negative for CD31, CD45, and CD34 (Fig. 1b), which is consistent with the characteristics previously reported [16].

To identify the multi-differentiation potential of the CDCs, permanent ligation of the left anterior descending coronary artery was performed to induce MI injury, and CDCs were intramyocardially injected into the infarct border zone. Immunostaining was performed 4 weeks post-transplantation. As shown in Additional file 1 (Figure S1), GFP staining was used to identify the transplanted CDCs, and a few CDCs were demonstrated to express tropomyosin, vWF, or α-SMA, indicating that CDCs could give rise to cardiomyocytes, endothelial cells, and smooth muscle cells, although the differentiation efficacies were very low.

### MET augmented the therapeutic effect of CDC transplantation against MI

CDCs were pretreated with MET at 100 μmol/L for 60 min before transplantation. The recipient wild-type C57 mice were randomly assigned to different experimental groups. MI was induced and 1 × 10^5^ CDCs were intramyocardially injected into the infarct border zone immediately after MI. MET treatment was induced by intraperitoneal injection of MET at a dose of 125 mg/kg/day for a week. The initial areas at risk (i.e., the area of ischemia) of the infarcted hearts in the different groups with MI injury were comparable (36.18 ± 1.73%; Additional file 2: Figure S2A). Echocardiography was performed 4 h after MI induction and showed that the post-MI mice had an equivalent reduction in EF% (Additional file 2: Figure S2B).

**Fig. 2:**
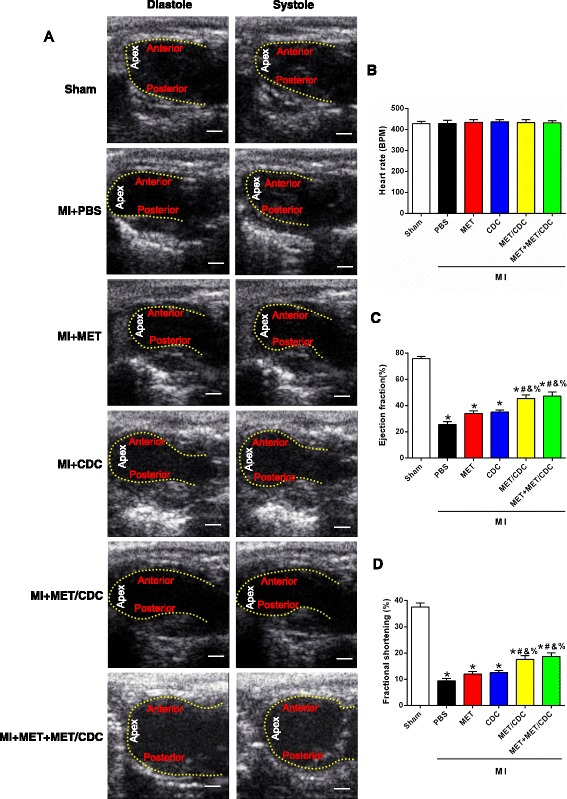
Combination of metformin (*MET*) treatment and cardiosphere-derived cell (*CDC*) transplantation improved cardiac function in myocardial infarction (*MI*) mice. **a** Representative echocardiographic images of hearts with different treatments at 4 weeks post-MI. *Scale bar* = 2 mm. **b** Heart rates were controlled to be similar in different groups. **c**,**d** Left ventricular ejection fraction and fraction shortening at 4 weeks post-MI. *n* = 8. Data were analyzed by one-way ANOVA with post-hoc comparisons by the Tukey’s test. *P < 0.05 vs. sham; ^#^ *P* < 0.05 vs. MI + phosphate-buffered saline (*PBS*); &*P* < 0.05 vs. MI + MET; ^%^ *P* < 0.05 vs. MI + CDC. *MET/CDC* MET-pretreated CDCs

**Fig. 3:**
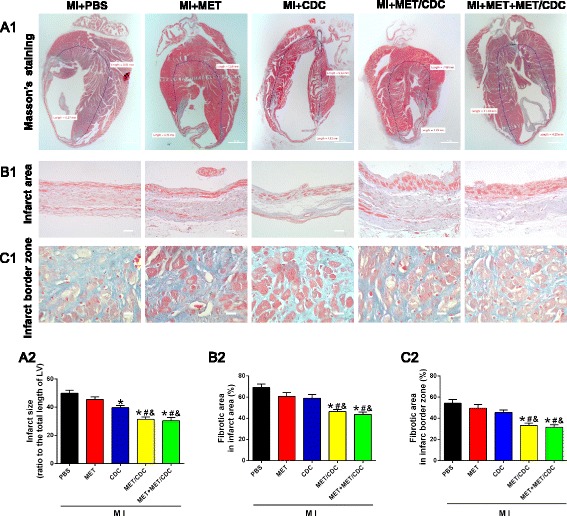
Combination of metformin (*MET*) treatment and cardiosphere-derived cell (*CDC*) transplantation reduced infarct size in myocardial infarction (*MI*) mice. **a1** Representative images of Masson’s trichrome staining for heart tissue obtained from hearts with different treatments at 4 weeks post-MI. *Scale bar* = 1 mm. **a2** Graphic representation of the left ventricular (*LV*) infarct size calculated as the ratio of midline length of the infarcted LV wall to the midline length of total LV wall (*n* = 5). **b** Representative images (**b1**) and quantification (**b2**) of the fibrotic area in the infarct area 4 weeks post-MI. *Scale bar* = 200 μm. **c** Representative images (**c1**) and quantification (**c2**) of the fibrotic area at the infarct border zone 4 weeks post-MI. *Scale bar* = 100 μm. *n* = 5. Data were analyzed by one-way ANOVA with post-hoc comparisons by the Tukey’s test. *P < 0.05 vs. MI + phosphate-buffered saline (*PBS*); ^#^ *P* < 0.05 vs. MI + MET; &*P* < 0.05 vs. MI + CDC. *MET/CDC* MET-pretreated CDC

At 4 weeks post-MI, treatment with CDCs or MET alone modestly improved the cardiac function. Transplantation with MET-pretreated CDCs, with or without MET injection, significantly improved the cardiac function compared with the other groups (Fig. 2). Meanwhile, Masson’s trichrome staining was used to measure infarct size and fibrosis. CDCs or MET treatment slightly reduced infarct size at 4 weeks post-MI. Transplantation with MET-pretreated CDCs, with or without MET injection, further reduced MI-induced infarct size (Fig. 3a). In addition, the percentage of fibrotic areas both in the total infarction and peri-infarct zone were significantly reduced in these two groups (Fig. 3b and c). These data suggested that MET pretreatment augmented the therapeutic effect of CDC transplantation against MI injury.

### MET pretreatment promoted CDC survival in post-MI heart

**Fig. 4:**
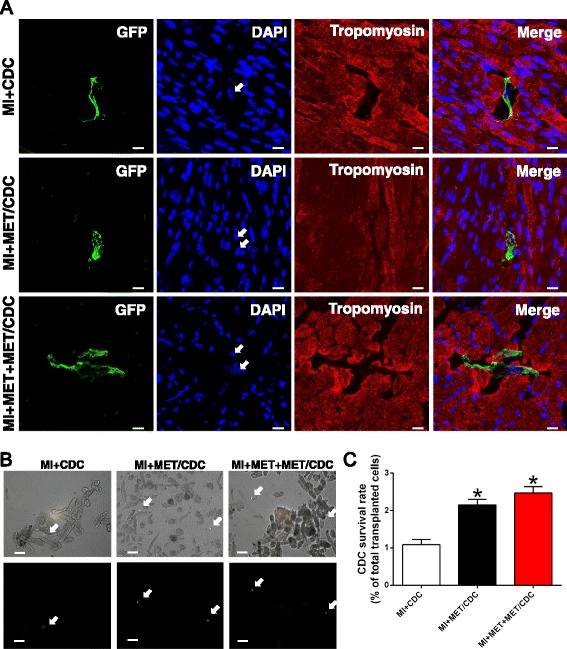
Metformin (*MET*) treatment increased cardiosphere-derived cell (*CDC*) survival after transplantation into post-myocardial infarction (*MI*) hearts. **a** Representative images of survived CDCs in hearts at 7 days after transplantation. Heart tissue was immunostained for tropomyosin (*red*), green fluorescent protein (*GFP*) (*green*) and DAPI (*blue*). *Scale bar* = 100 μm. **b** Post-MI hearts with CDC transplantation were enzymatically digested, and small cells from the heart (<30 μm diameter) were collected after depletion of cardiomyocytes. As indicated with the *white arrows*, GFP-positive cells represent surviving CDCs under fluorescence microscope. *Scale bar* = 50 μm. **c** The percentage of surviving CDCs out of the total transplanted CDCs at 7 days post-transplantation. *n* = 5. Data were analyzed by one-way ANOVA with post-hoc comparisons by the Tukey’s test. *P < 0.05 vs. MI + CDC. *MET/CDC* MET-pretreated CDC

The heart tissue was harvested at different time points after CDC transplantation. Immunostaining was performed to identify the surviving CDCs with GFP labeling. As shown in Fig. 4a, the GFP^+^ CDCs were observed in heart tissue 7 days post-transplantation, and significantly more GFP^+^ cells were seen in the MET-pretreated groups. It is known that death of stem cells predominantly occurs within the first 24 h post-transplantation into the myocardium. Thus, the hearts were enzymatically digested at 24 h post-transplantation and the GFP^+^ cells were carefully quantified. CDCs survived more when pretreated with MET before transplantation, and a combination of intraperitoneal administration of MET further increased the CDC survival (survival rates of 1.08 ± 0.14% vs. 2.15 ± 0.15% vs. 2.46 ± 0.17%, for MI + CDC, MI + MET/CDC, and MI + MET + MET/CDC, respectively; *n* = 4; *P* < 0.05) (Fig. 4b and c).

### Effects of MET-pretreated CDC transplantation on post-MI myocardial inflammation

**Fig. 5:**
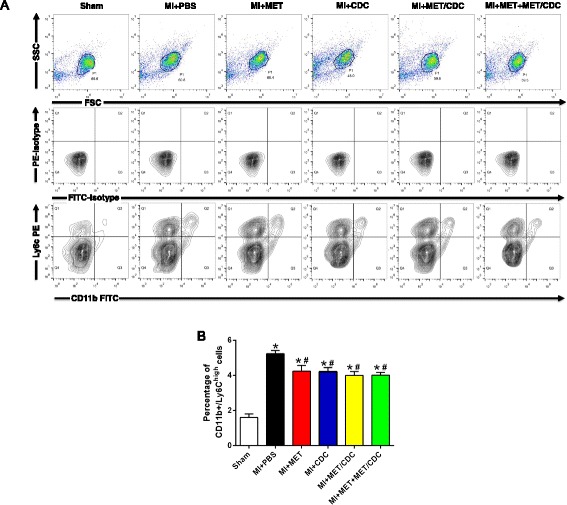
The effect of metformin (*MET*) treatment on the inflammatory response in post-myocardial infarction (*MI*) hearts. Representative contour plots (**a**) and quantification (**b**) of infiltrating monocyte population (Ly6C^high^/CD11b^+^) in cells isolated from infarcted hearts at 48 hours post-MI analyzed by FACS analysis. *n* = 5. Data were analyzed by one-way ANOVA with post-hoc comparisons by the Tukey’s test. *P < 0.05 vs. Sham; ^#^ *P* < 0.05 vs. MI + phosphate-buffered saline (*PBS*). *MET/CDC* MET-pretreated cardiosphere-derived cells

We next investigated the inflammatory cell infiltration using flow cytometry at 48 h after MI. The percentage of CD11b^+^/Ly6C^high^ cells was robustly upregulated by MI injury, which was reduced by MET treatment or CDC transplantation. However, the combination of MET and CDC transplantation did not exert stronger anti-inflammatory effects (Fig. 5), suggesting that the enhanced CDC survival and function induced by MET might not be associated with the inflammatory response.

### MET attenuated oxidative stress-induced CDC apoptosis via AMPK activation

**Fig. 6:**
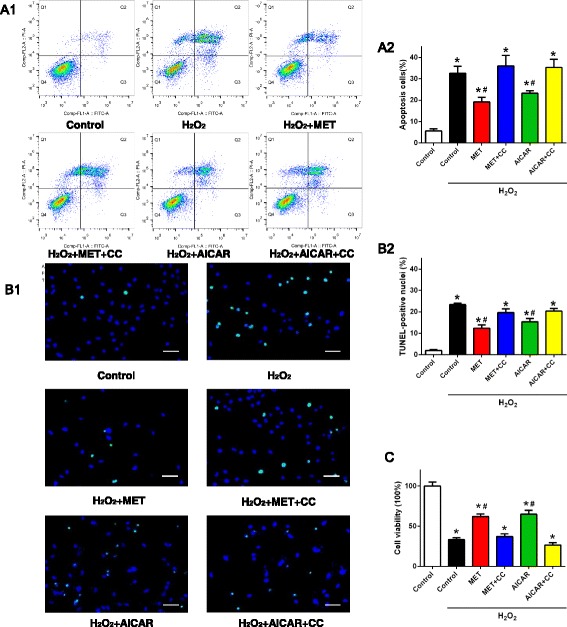
Metformin (*MET*) treatment exerted an anti-apoptotic effect on CDCs subjected to oxidative stress by activating AMPK. **a1** Annexin V-PI double staining assay of CDCs treated with H_2_O_2_, analyzed by flow cytometric analysis. **a2** Average percentages of apoptotic CDCs. *n* = 9. **b** Representative images (**b1**) and quantification (**b2**) of TUNEL staining in CDCs subjected to H_2_O_2_ treatment. Cell nuclei were stained with DAPI (*blue*) and TUNEL^+^ nuclei were labeled with TMR (*green*). TUNEL positive rate = (TUNEL-positive nuclei/DAPI-positive nuclei) × 100%. *n* = 12. *Scale bar* = 30 μm. **c** Cell viability of CDCs detected with CCK8 analysis. The data were normalized to 100% of the control group. Data were analyzed by one-way ANOVA with post-hoc comparisons by the Tukey’s test. *P < 0.05 vs. control; ^#^ *P* < 0.05 vs. H_2_O_2_. *CC* compound C

Oxidative stress is known to be a major factor causing stem cell apoptosis in the transplantation microenvironment of the post-MI heart. CDCs were treated with 30 μmol/L H_2_O_2_ for 24 h to induce oxidative stress. CDC apoptosis was detected with TUNEL staining and flow cytometric analysis following annexin V and PI staining. CDC viability was measured with cell counting kit-8 (CCK8) assay. As shown in Fig. 6a and b, H_2_O_2_ induced CDC apoptosis and decreased CDC viability. MET pretreatment (100 μmol/L for 60 min) significantly decreased the TUNEL^+^ CDCs and annexin V^+^/PI^–^ CDCs subjected to H_2_O_2_ insult. The decreased CDC viability induced by H_2_O_2_ was also largely restored by MET (Fig. 6c). When the AMPK inhibitor compound C (CC; 20 μmol/L) was used to pretreat the CDCs 30 min before MET administration, the anti-apoptotic effect of MET was blunted. The AMPK activator AICAR also reduced H_2_O_2_-induced CDC apoptosis, which was blocked by CC pretreatment (Fig. 6a and b). These data suggest that MET attenuated oxidative stress-induced CDC apoptosis via activation of AMPK.

### AMPK/eNOS signaling pathway mediated the pro-survival effect of MET on CDCs

**Fig. 7:**
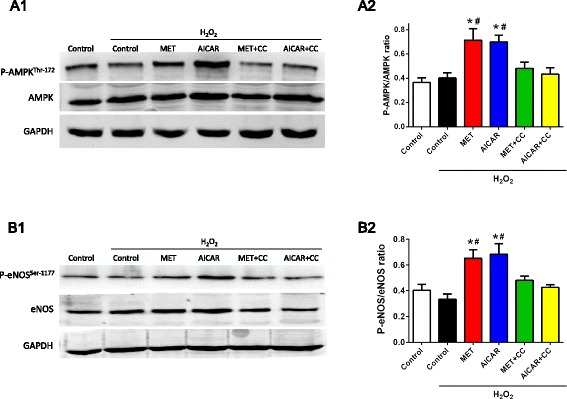
Metformin (*MET*) treatment increased phosphorylation of AMP-activated protein kinase (*AMPK*)/endothelial nitric oxide synthase (*eNOS*) in CDCs. Representative blots (**a1**) and quantification (**a2**) of AMPK phosphorylation. AMPK phosphorylation was represented as the ratio of phosphorylated α-AMPK at Thr-172 to total AMPK of CDCs. Representative blots (**b1**) and quantification (**b2**) of eNOS phosphorylation. eNOS phosphorylation was represented as the ratio of phosphorylated eNOS at Ser-1177 to total eNOS of CDCs. Data were analyzed by one-way ANOVA with post-hoc comparisons by the Tukey’s test. *P < 0.05 vs. control; ^#^ *P* < 0.05 vs. H_2_O_2_. *CC* compound C

To further determine how MET resulted in AMPK activation, α-AMPK phosphorylation in CDCs was evaluated with Western blotting analysis. The total AMPK protein expression was comparable among each experimental group. Compared with control, H_2_O_2_ treatment did not change the expression of phosphorylated α-AMPK at Thr-172 in CDCs. The level of phosphorylated α-AMPK significantly increased when CDCs were treated with MET or AICAR, which was blocked by pretreatment with the AMPK inhibitor CC (Fig. 7a). As the downstream target of AMPK, eNOS is known to regulate cell apoptosis. Our data show that the level of phosphorylated eNOS at Ser-1177 expression was slightly decreased by H_2_O_2_ treatment. MET and AICAR significantly increased the eNOS phosphorylation, which was also blocked by pretreatment with CC (Fig. 7b). These data suggest that the MET-induced pro-survival effect against oxidative stress might be associated with the AMPK/eNOS signaling pathway.

## Discussion

Much progress has been made in the field of stem cell transplantation for treating MI, but challenges such as poor cell survival in the harsh transplantation microenvironment greatly limit its therapeutic effect. In this study, we combined MET treatment and CDC transplantation for treating MI injury in mice, and found that MET improved CDC survival thereby enhancing its therapeutic effect after transplantation. Our data showed that the anti-apoptotic function of MET on CDCs was associated with AMPK activation and eNOS phosphorylation. Since MET is a safe medication in clinical practice, combination of MET treatment with stem cell therapy is of clinical significance to treat MI patients.

The fact that CDC transplantation elicits cardioprotective effects has been shown in small and large animals, and in patients with MI injury. Aside from autologous CDC transplantation, ‘off-the-shelf’ allogeneic human CDCs are being tested clinically in the ALLSTAR and DYNAMIC trials [17]. CDCs have multi-differentiation capacity in vivo, which was confirmed in our study. It is also known that the therapeutic effect of CDCs is mainly mediated by the paracrine effect rather than by direct differentiation [18, 19]. CDCs were reported to stimulate increased myocyte proliferation compared with other stem cell types such as bone marrow-derived mesenchymal stem cells [20], probably because CDCs exhibited a balanced profile of paracrine factor production [21]. The effects of both the paracrine effect and differentiation rely on the quantity of the surviving CDCs. In our study, we speculate that these functions of CDCs were improved when more post-transplanted CDCs survived with MET treatment. The reduced infarct size might be due to decreased myocyte apoptosis and increased new cardiomyocyte formation.

Despite the progress in recent years, the survival of transplanted cells in infarcted myocardium is still poor [5, 22]. Previous studies reported that only <5% of post-transplanted stem cells survive in the peri-infarct area. Factors such as ischemia, oxidative stress, and inflammation hamper stem cell survival in the post-MI heart. MET is reported to attenuate inflammation [11], but our data indicated that the pro-survival effect of MET on CDCs in the infarcted heart was less likely associated with its influence on inflammation. Oxidative stress alters the balance of the production and elimination of intracellular oxygen-free radicals and results in over-accumulation of reactive oxygen species (ROS), which are major factors inducing the apoptosis of transplanted stem cells [23]. Efforts including modification of cell survival-associated genes have been made [5], but these methods are far from clinical use. We demonstrated that MET pretreatment exerted an anti-apoptotic effect on CDCs subjected to oxidative stress in vitro, and that MET promoted the survival of post-transplantation CDCs in vivo. The combination of MET and CDC transplantation exerted a significantly greater effect on increasing cardiac function and reducing infarct size of the infarcted heart, indicating that MET is a ready-to-use medication for augmenting the therapeutic effect of stem cell transplantation.

MET is recognized to activate AMPK, which is essential for keeping the balance of energy production and metabolism in various tissues, thereby modulating cell apoptosis. MET can also exert cardioprotective effects independently of anti-hyperglycemic effects. MET-induced AMPK activation exerts protective effects against acute oxidative stress-induced injury in cardiomyocytes [24]. AMPK works as a protein kinase to activate eNOS by phosphorylating eNOS at Ser-1177 [25]. As a potent protective signaling molecule, eNOS is expressed in vascular endothelial cells, cardiomyocytes, and different types of stem cells. Overexpression of eNOS generates low amounts of NO, which regulates the apoptosis of embryonic stem cells [26]. MET improves cardiomyocyte survival under ischemia/perfusion injury, which is associated with increases in AMPK and eNOS phosphorylation [13]. We demonstrated that MET increased AMPK and eNOS phosphorylation in CDCs. Another AMPK activator, AICAR, showed similar effects to MET. These data suggest that the MET-induced anti-apoptotic effect on CDCs might be associated with the AMPK/eNOS pathway.

## Conclusions

Our study reveals that MET treatment significantly enhances the therapeutic effect of CDC transplantation against MI injury. The pro-survival effect of MET on CDCs might be through an AMPK-eNOS-dependent mechanism. Since MET is a safe and ready-to-use medication, combination of MET treatment and stem cell therapy may be a promising strategy in clinical practice.

## Additional files

Additional file 1: Figure S1. The CDC differentiation at 4 weeks after transplantation analyzed by immunostaining. A–C: Sections of hearts were immunostained with antibodies to (A) the cardiomyocyte marker tropomyosin, (B) the endothelial cell marker von-Willebrand Factor (vWF), and (C) the smooth muscle cell marker α-smooth muscle actin (α-SMA). Antibody to GFP was used for identifying surviving CDC-derived cells and DAPI was used for identifying nuclei. *Scale bars* = 20 μm. *DAPI* 4′,6-diamidino-2-phenylindole. (PDF 178 kb)
Additional file 2: Figure S2. (A1) Representative images for area at risk (AAR) of mice hearts 4 h post-MI. (A2) Quantitative data for AAR and area not at risk (ANAR) of mice hearts 4 h post-MI. (B) Averaged ejection fraction (EF) assessed by echocardiography of mice hearts 4 h post-MI. (PDF 152 kb)
