# Correction: Metformin promotes the survival of transplanted cardiosphere-derived cells thereby enhancing their therapeutic effect against myocardial infarction

**DOI:** 10.1186/s13287-022-03224-0

**Published:** 2022-12-23

**Authors:** Rongchuan Yue, Wenbin Fu, Xiang Liao, Cong Lan, Qiao Liao, Liangpeng Li, Dezhong Yang, Xuewei Xia, Xiongwen Chen, Chunyu Zeng, Wei Eric Wang

**Affiliations:** 1 Department of Cardiology, Daping Hospital, Chongqing Institute of Cardiology, Third Military Medical University, 10 Changjiangzhilu Road, Yuzhong District, Chongqing, 400042 China; 2 Department of Cardiology, Chuanbei Medical College, Sichuan, 637007 China

**Correction: Stem Cell Research & Therapy (2017) 8:17** https://doi.org/10.1186/s13287-017-0476-7

When sorting out the original data, the authors noted the representative masson images for MI + MET/CDC and MI + MET + MET/CDC groups in Fig. 3C1 were mistakenly uploaded when assembling the figure. The masson images for MI + MET/CDC and MI + MET + MET/CDC groups were incorrect. To make sure the colors of re-photographed masson images were consistent, the images of all groups in Fig. 3C1 were changed. Although this does not affect the final conclusion, we are still sorry for our mistake. Figure 3C1 has now been updated [1].

**Fig. 3:**
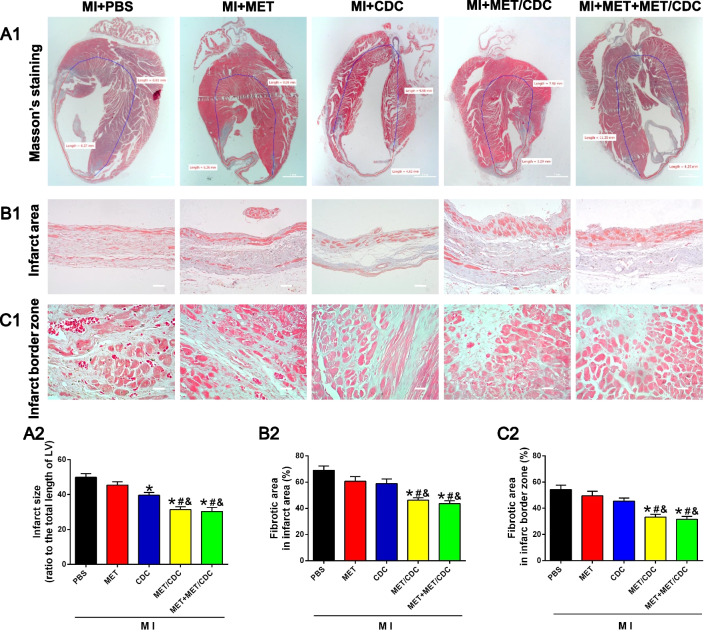
Combination of metformin (*MET*) treatment and cardiosphere-derived cell (*CDC*) transplantation reduced infarct size in myocardial infarction (*MI*) mice.** a1** Representative images of Masson’s trichrome staining for heart tissue obtained from hearts with different treatments at 4 weeks post-MI. Scale bar = 1 mm.** a2** Graphic representation of the left ventricular (*LV*) infarct size calculated as the ratio of midline length of the infarcted LV wall to the midline length of total LV wall (*n* = 5).** b** Representative images (**b1**) and quantification (**b2**) of the fibrotic area in the infarct area 4 weeks post-MI. Scale bar = 200 μm.** c** Representative images (**c1**) and quantification (**c2**) of the fibrotic area at the infarct border zone 4 weeks post-MI. Scale bar = 100 μm. n = 5. Data were analyzed by one-way ANOVA with post-hoc comparisons by the Tukey’s test. *P < 0.05 vs. MI + phosphate-buffered saline (PBS); #* P* < 0.05 vs. MI + MET; &*P* < 0.05 vs. MI + CDC.* MET/CDC* MET-pretreated CDC
