# Supplementary figures and images for: Metformin promotes the survival of transplanted cardiosphere-derived cells thereby enhancing their therapeutic effect against myocardial infarction
Source: Stem Cell Res Ther. 2017 Jan 28;8:17. doi: 10.1186/s13287-017-0476-7 (PMC5273815; doi:10.1186/s13287-017-0476-7)

Supplementary Figure 1

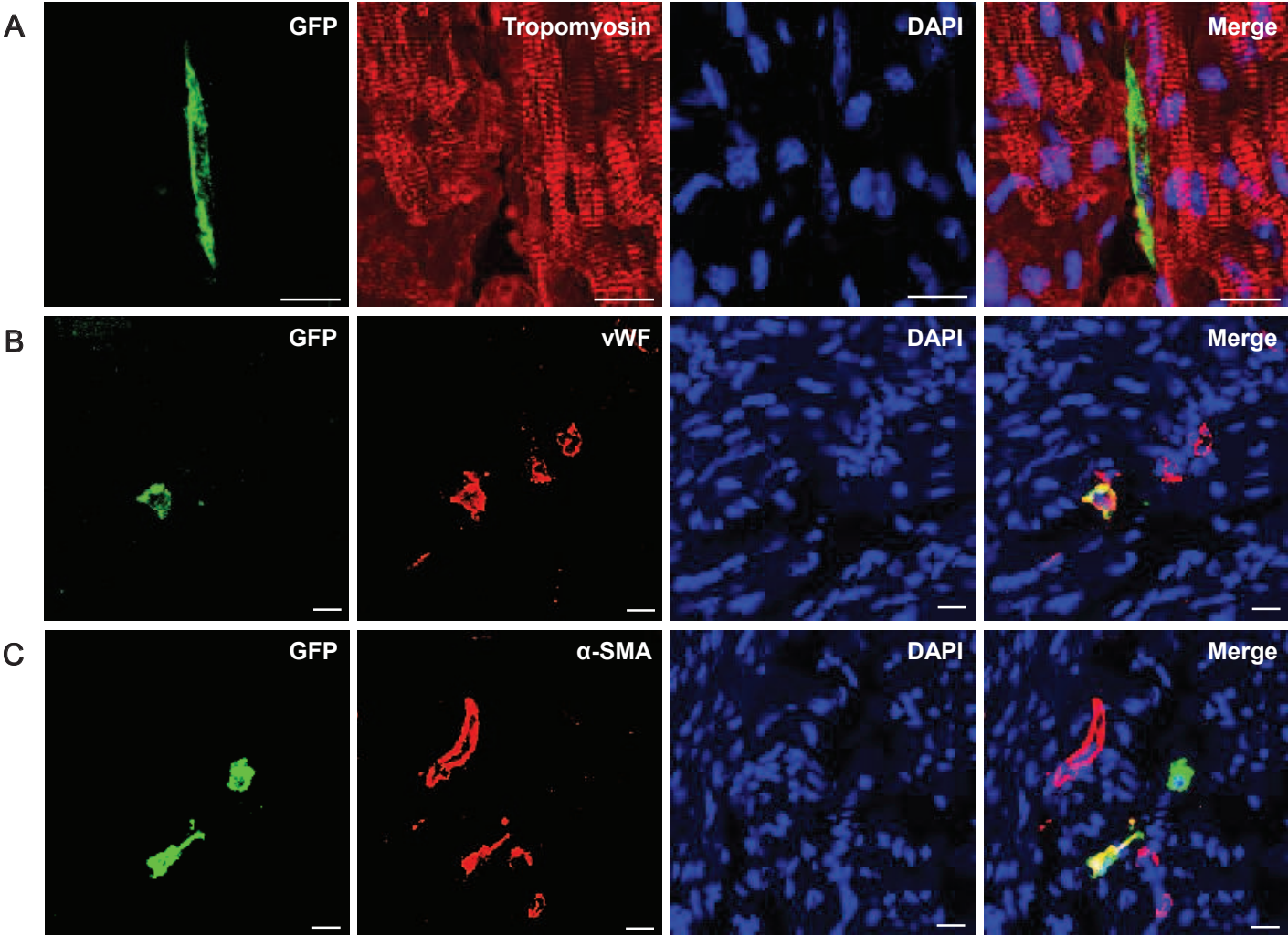

Supplement: Additional file 1: Figure S1. — The CDC differentiation at 4 weeks after transplantation analyzed by immunostaining. A–C: Sections of hearts were immunostained with antibodies to (A) the cardiomyocyte marker tropomyosin, (B) the endothelial cell marker von-Willebrand Factor (vWF), and (C) the smooth muscle cell marker α-smooth muscle actin (α-SMA). Antibody to GFP was used for identifying surviving CDC-derived cells and DAPI was used for identifying nuclei. Scale bars = 20 μm. DAPI 4′,6-diamidino-2-phenylindole. (PDF 178 kb) [file 13287_2017_476_MOESM1_ESM.pdf]

Supplementary Figure 2

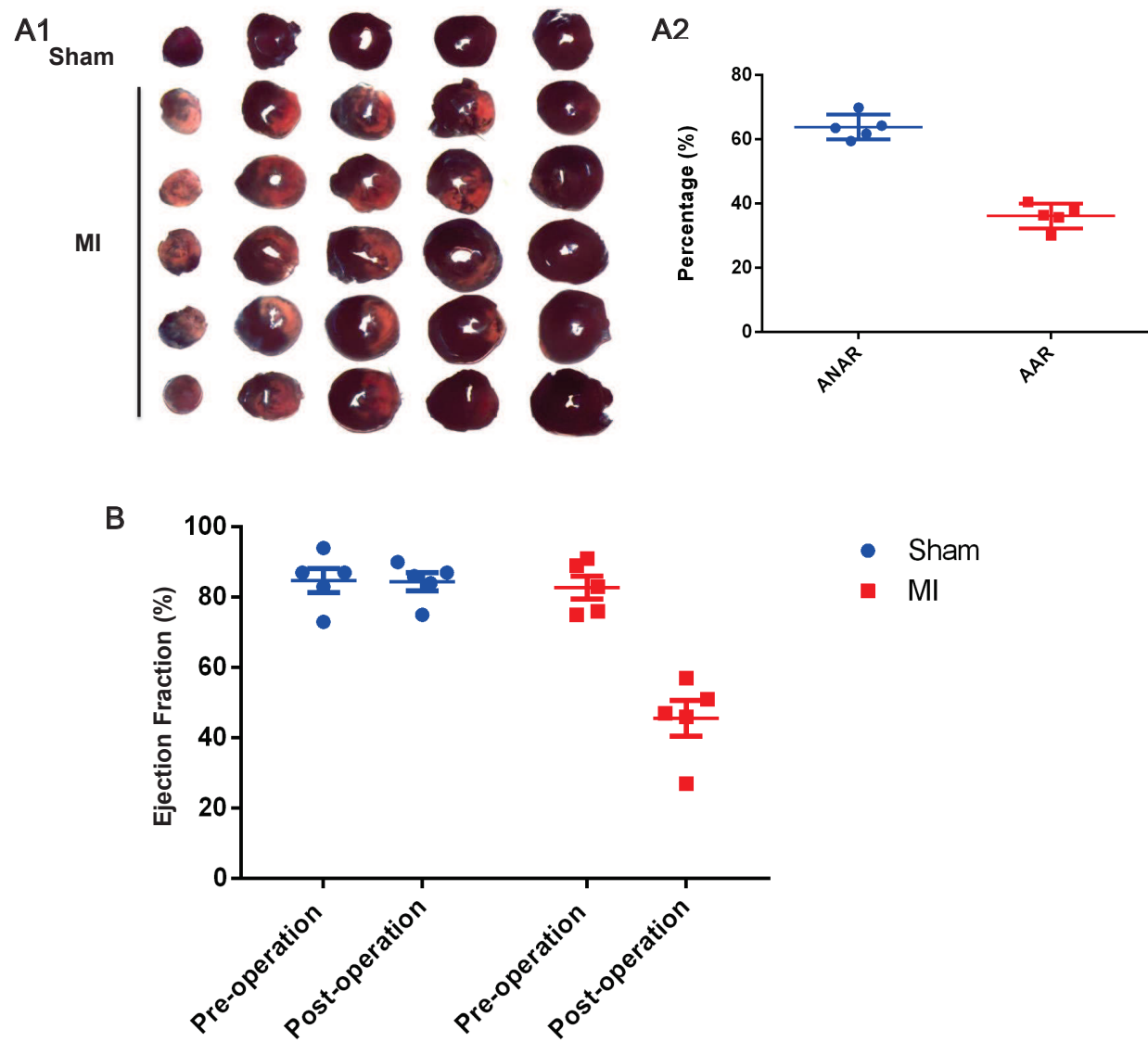

Supplement: Additional file 2: Figure S2. — (A1) Representative images for area at risk (AAR) of mice hearts 4 h post-MI. (A2) Quantitative data for AAR and area not at risk (ANAR) of mice hearts 4 h post-MI. (B) Averaged ejection fraction (EF) assessed by echocardiography of mice hearts 4 h post-MI. (PDF 152 kb) [file 13287_2017_476_MOESM2_ESM.pdf]
